# Drivers of informal sector and non-prescription medication use in pediatric populations in a low- and middle-income setting: A prospective cohort study in Zambia

**DOI:** 10.1371/journal.pgph.0002072

**Published:** 2023-07-06

**Authors:** Sanne Wildbret, Logan Stuck, Chaluma C. Luchen, Michelo Simuyandi, Caroline Chisenga, Constance Schultsz, Vanessa C. Harris

**Affiliations:** 1 Amsterdam Institute for Global Health and Development, Amsterdam, The Netherlands; 2 Department of Global Health, Amsterdam UMC, Location University of Amsterdam, Amsterdam, The Netherlands; 3 Centre for Infectious Disease Research in Zambia, Lusaka, Zambia; 4 Department of Medical Microbiology, Amsterdam UMC, Location University of Amsterdam, Amsterdam, The Netherlands; 5 Amsterdam Institute for Infection and Immunity, Infectious Diseases, Amsterdam, The Netherlands; 6 Division of Infectious Diseases, Department of Internal Medicine, Amsterdam UMC, Location University of Amsterdam, Amsterdam, The Netherlands; Institute of Microbial Technology CSIR, INDIA

## Abstract

Obtaining medication from the informal sector is common in low- and middle- income countries. Informal sector use increases the risk for inappropriate medication use, including inappropriate antibiotic usage. Infants are at the highest risk of complications from inappropriate medication use, yet there is insufficient knowledge about the risk factors driving caregivers to obtain medication from the informal sector for young children. We aimed to define infant and illness characteristics associated with use of medication purchased in the informal sector for infants up to fifteen months of age in Zambia. We used data from, a prospective cohort study (ROTA-biotic) conducted among 6 weeks to 15 months old children in Zambia, which is nested within an ongoing phase III rotavirus vaccine trial (Clinicaltrial.gov NCT04010448). Weekly in-person surveys collected information about illness episodes and medication usage for the trial population and for a community control cohort. The primary outcome for this study was whether medication was purchased in the formal sector (hospital or clinic) or informal sector (pharmacy, street vendor, friend/relative/neighbor, or chemical shop) per illness episode. Descriptive analyses were used to describe the study population, and the independent and medication use variables stratified by the outcome. A mixed-effects logistic regression model with a participant-level random intercept was used to identify independent variables associated with the outcome. The analysis included 439 participants accounting for 1927 illness episodes over fourteen months in time. Medication was purchased in the informal sector for 386 (20.0%) illness episodes, and in the formal sector for 1541 (80.0%) illness episodes. Antibiotic usage was less common in the informal sector than in the formal sector (29.3% vs 56.2%, p < 0.001, chi-square). Most medications purchased in the informal sector were orally administered (93.4%), and non-prescribed (78.8%). Increased distance from the closest study site (OR: 1.09; 95% CI: 1.01, 1.17), being included in the community cohort site (OR: 3.18; 95% CI: 1.86, 5.46), illnesses with general malaise fever, or headache (OR: 2.62; 95% CI: 1.75, 3.93), and wound/skin disease (OR: 0.36; 95% CI: 0.18, 0.73) were associated with use of medication from the informal sector. Sex, socioeconomic status, and gastrointestinal disease were not associated with use of medication from the informal sector. Informal sector medication use is common and, in this study, risk factors for obtaining medications in the informal sector included a long distance to a formal clinic, type of illness, and not being enrolled in a clinical trial. Continued research on medication use from the informal sector is crucial and should include generalizable study populations, information on severity of disease, emphasis on qualitative research, and a move towards testing interventions that aim to improve access to formal health care settings. Our findings suggest that improved access to formal health care services may decrease reliance on medication from the informal sector for infants.

## Introduction

In low- and middle-income countries (LMIC), informal healthcare makes up a large component of health care provision, particularly for low-income individuals [1]. Caregivers in Sub-Saharan Africa often acquire medication for their infants from the informal health sector [2], increasing the risk of inappropriate antibiotic usage and placing infants at higher risk for complications from inappropriate drug use.

Care provision by the informal health sector is often of poor quality, particularly for evidence-based medication prescribing practices, caused by profit incentives, underqualified staff and an increase in the trade of substandard and counterfeit medicines leading to treatment failures [2–4]. Use of medication from the informal health sector also contributes to inappropriate antibiotic usage and the development of antibiotic resistance, with unregulated antibiotics being readily available in shops and drug stores [5–7].

Infants are at particularly high risk of complications from drug use obtained in the informal health sector since they are more vulnerable to medication dosing errors and lack communication skills [8, 9]. Caregivers frequently (71%) estimate medication dosages for young children, and in 58% of cases caregivers make a dosage error when medicating their children, yet only half of parents are aware of the risks of self-medication [10, 11]. In addition, consulting the informal sector for medication might delay a child from receiving appropriate care in the formal sector [10, 12].

It is therefore necessary to understand the determinants of informal healthcare usage in LMICs to decrease informal health care reliance. Healthcare providers and policy makers need data on how best to reduce barriers to regulated healthcare for infants and best prevent misuse and overuse of medication in the informal sector. The current research aims to identify infant and illness characteristics associated with use of medication purchased in the informal sector by infants up to fifteen months of age in Zambia.

## Methods

### Study design

The current research utilizes data from the ongoing ROTA-biotic study. ROTA-biotic is a prospective cohort study nested within an ongoing phase III double-blind multinational randomized controlled trial assessing the safety, immunogenicity, and efficacy of a novel trivalent parenteral rotavirus vaccine as compared to the oral vaccine, Rotarix^™^, in the prevention of severe rotavirus gastroenteritis in healthy infants up to the age of 2 years (Clinicaltrials.gov NCT04010448). The ROTA-biotic study added an additional endpoint to this clinical trial, examining the efficacy of rotavirus vaccination (RVV) in preventing antibiotic consumption in enrolled infants by collecting data on medication usage among participants and adding a community control cohort.

### Study setting and population

The ROTA-biotic study is being conducted in Zambia and Ghana. This study uses data available from the study population in Zambia. Participants were recruited in three peri-urban communities in Lusaka district, namely: Matero, George and Chainda served by three clinical research sites. The ROTA-biotic study commenced in the second quarter of 2021 and is expected to run until the fourth quarter of 2023.

The ROTA-biotic study has two arms, the vaccinated cohort (consisting of infants also enrolled in the Phase III vaccine trial) and a community control cohort (to measure background incidence of antibiotic use). Infants in the vaccinated cohort are enrolled at study clinics in George and Matero. Infants in the community cohort are recruited using the same recruitment and follow-up strategies as the parent trial but are from a community away from the vaccine trial sites, which is Chainda. The ROTA-biotic study follows infants from 1.5 months until two years of age. Our study includes a study population ranging from 1.5 to 15 months of age.

### Community sensitization and recruitment

Mothers were informed about the study during their pregnancy visit or first baby visit to the community clinic. They were invited to enroll during the vaccination visit when the baby is 6 weeks old.

### Enrollment, inclusion, and exclusion

This study uses the preliminary data from both cohorts of the ROTA-biotic study in Zambia that was available through July 2022. The ROTA-biotic inclusion-criteria are healthy infants between 6 and 8 weeks of age at the time of inclusion, whose parent is able and willing to provide written informed consent and intends to stay in the same area with the child during the study period. ROTA-biotic exclusion criteria included concurrent participation in another clinical trial, presence of severe malnutrition or a systemic disorder, history of premature birth and/or birth weight of less than 2.5 kg, history of congenital abdominal disorders, major congenital or genetic defect, and participant’s parent not being available or willing to engage in active weekly follow-up. Participants that suffered from acute disease at the time of enrollment, or that experienced fever (>37.6° C) on the day of enrollment were temporarily excluded from participating in the study.

This study only included data from a participant from the ROTA-biotic study if the participant experienced at least one illness episode where medication was used during the study period. The data on medication use was provided by the caregiver. Illness episodes for which information on any of the variables of interest was not available were excluded due to missing data.

### Data collection

Data were collected by research assistants through interviewer-administered questionnaire using electronic case report forms (eCRF’s). The research assistants were experienced and received training and refreshment courses. ODK Collect software was used for online data entry via tablets. Four eCRF’s were used: enrollment, initial community visit (enrollment eCRF for community cohort arm participants), initial household visit, and weekly follow-up. During the weekly follow-up visit data on medication use was gathered by a medicine cabinet survey. Parents were asked to save prescriptions and wrappers of medications used, which were reviewed by the trained research assistants. If an enrolled infant was hospitalized, hospital antibiotic usage was also collected by the trained research assistant. The trained research assistant went to the hospital to collect data on hospital antibiotic usage and often accompanied participants to the hospital.

### Measures

The primary outcome is whether medication is purchased in the informal sector (pharmacy, street vendor, friend/relative/neighbor, or chemical shop) or formal sector (hospital or clinic). We ranked pharmacy as informal health sector since most medications acquired at the pharmacy were non-prescribed (83.5%) (S1 Table). To account for possible prescription usage in pharmacies, a secondary analysis included pharmacies within the formal health sector. Our data were organized in three levels: participant level, illness episode level, and medication use episode level. The primary outcome was analyzed at illness episode level. Independent variables included sex, study site, SES, distance to the closest study site, and type of illness. Medication-use related variables included antibiotic usage, number of medications used, route of administration, and prescription. We analyzed route of administration and prescription on a medication use episode level.

### Data processing and analysis

For statistical analyses the data were exported to RStudio [13]. A descriptive analysis was performed to summarize the sociodemographic characteristics of the study population. Electrical supply was included as an indicator of SES as compared to the general population of Zambia. The Euclidean distance between household and closest study site was calculated using GPS coordinates from the study sites and the household. Frequencies and percentages for all independent variables and medication use related variables stratified by use of medication from the formal and informal sector, and the P-value for chi-square test were calculated.

A mixed-effects logistic regression model was constructed to identify independent variables associated with the dependent variable. The model includes a participant-level random intercept to account for a subject-specific baseline probability of informal drug usage. The SES relative to the rest of the study population was calculated for every participant by performing a principal components analysis (PCA) based on data from the initial household visit on water source and availability, toilet facility, ownership of animals and/or agricultural land, structural condition of the house, electrical supply, transport facilities, and available electronics and appliances. The first component of the PCA was used as the SES index value [14]. The variables number of medications per illness episode, antibiotic usage, route of administration, and prescription were not included in the mixed effects model, since the causal relation remains unclear.

### Ethical considerations

For the ROTA-biotic study ethical approval was obtained from both the University of Zambia Biomedical Research Ethics Committee (1100–2020) and the Zambian National Health Research Authority.

## Results

### Study population

**Fig 1:**
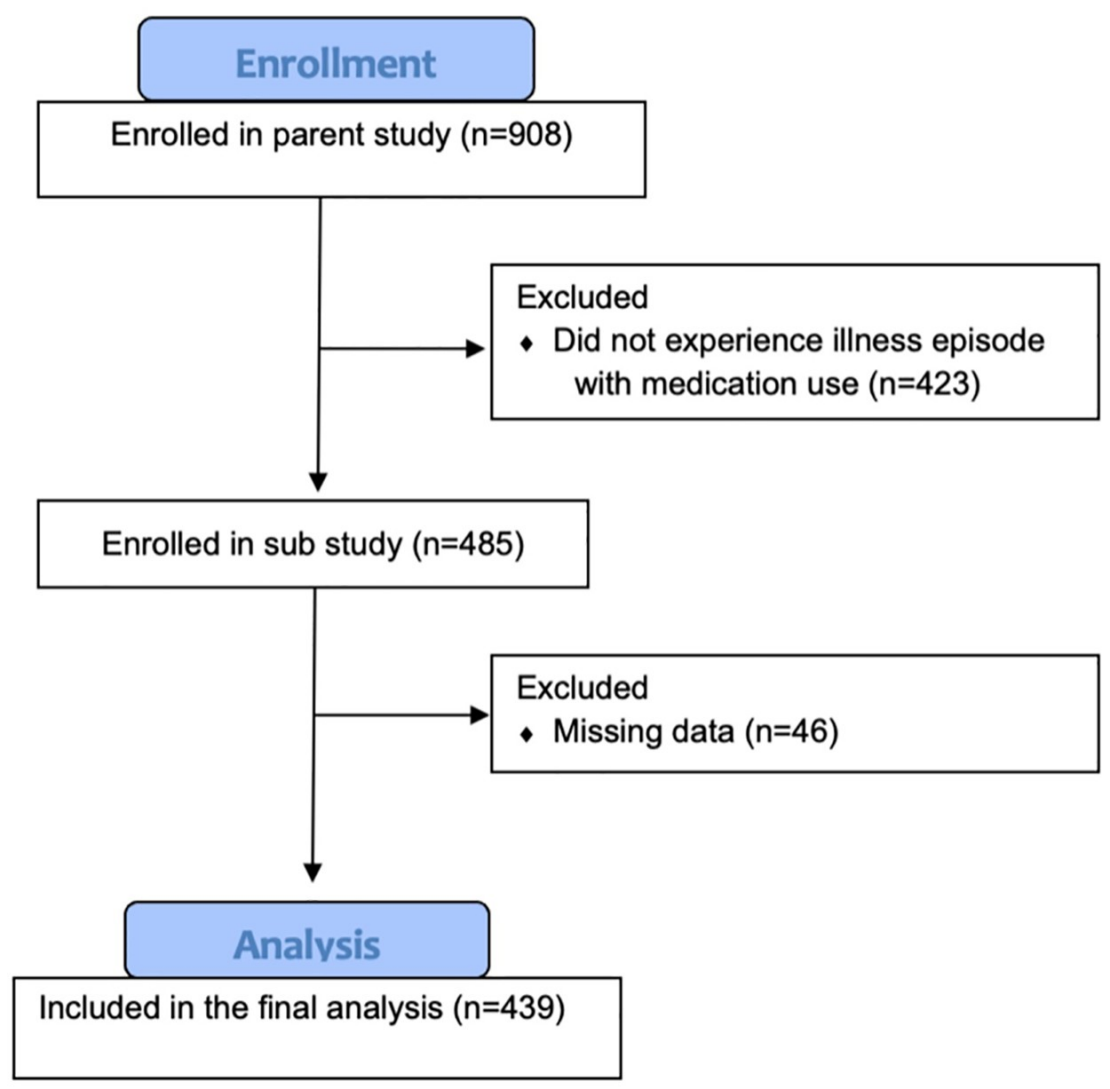
CONSORT flow diagram of participants included in the analysis.

As of July 2022, data from 908 participants were available from the ROTA-biotic study, consisting of 19,821 weekly follow-up visits and 2,883 illness episodes. Including participants who experienced at least one illness episode during which medication was used, and controlling for missing data, 439 participants were included in the analysis, accounting for 1,927 illness episodes over 14 months (Fig 1). There was a similar proportion of males and females in the study (Table 1; 46.7% vs 53.3%, respectively). Participants were distributed unequally across study sites, with the largest proportion of participants from George (53.1%), followed by the Chainda (community control site) (25.1%) and Matero (21.9%) study sites (Table 1). The largest proportion of participants, consisting of 370 (84.3%) participants, had access to either their own or a shared electricity connection, and 69 (15.7%) participants were without electrical supply (Table 1). Most participants lived close to the study sites, with 345 (78.6%) participants living within a 3-kilometer radius.

**Table 1:** Sociodemographic characteristics of the study population.

Variable	Category	Total N = 439 (100%)
Sex	Male	205 (46.7%)
	Female	234 (53.3%)
Study site	Matero	96 (21.9%)
	George	233 (53.1%)
	Chainda	110 (25.1%)
Identity	Ngoni	40 (9.1%)
	Bemba	121 (27.6%)
	Chewa	77 (17.5%)
	Lozi	9 (2.1)
	Tonga	40 (9.1%)
	Other	152 (34.6%)
Electrical supply	Own connection	135 (30.8%)
	Shared connection	235 (53.5%)
	No connection	69 (15.7%)
Distance to closest study site (km)	<1	169 (38.5%)
	≥1 and <3	176 (40.1%)
	≥3	94 (21.4%)

### Illness episode characteristics associated with informal sector medication use

Medications that were acquired within the informal sector were mostly acquired in pharmacies (62.4%), followed by over the counter/chemical shops (29.1%) (S2 Table). Numerous illness episode characteristics were associated with purchase of medication in the informal sector as compared to the formal sector (Table 2). Participants from the community cohort group in Chainda visited the informal health sector more often (35.4% of illness episodes) than trial participants (Matero (17.0%), and George (14.0%), p < 0.001, chi-square). SES was not associated with informal sector use, as infants living in a household with a high SES received their medication from the informal sector about as often as infants living in a household with a medium or low SES, 133 (20.7%) vs. 131 (20.3%) and 122 (19.1%) illness episodes, respectively (p = 0.746, chi-square). The proportion of illness episodes for which medication were acquired in the informal health sector increased with increasing distance from the study clinic, from 15.0% for <1 km to 22.0% for ≥3 km (Table 2; p < 0.001, chi-square). Most illness episodes were classified as respiratory disease (n = 1123), followed by gastrointestinal disease (n = 391). Acquisition of medication in the informal sector was more frequent for illness episodes categorized as ‘malaise, fever, or headache’ as opposed to other illness categories (40.5% vs 7.3%-20.6%; p < 0.001, chi-square). A secondary evaluation assessed illness episode characteristics when pharmacies were considered part of the formal sector rather than the informal sector (S4 Table) with similar associations.

**Table 2:** Illness episode-level participant and illness characteristics stratified by use of medication from the formal and informal sector.

Variable	Category	Total	Formal sector n (row %)	Informal sector n (row %)	P-value for chi-square test
**Illness episodes**		**N = 1927**	**1541 (80.0%)**	**386 (20.0%)**	
Sex	Male	N = 1000	794 (79.4%)	206 (20.6%)	0.555
	Female	N = 927	747 (80.6%)	180 (19.4%)	
Study site*	Matero	N = 429	356 (83.0%)	73 (17.0%)	<0.001
	George	N = 915	873 (86.0%)	142 (14.0%)	
	Chainda	N = 483	312 (64.6%)	171 (35.4%)	
SES	Low	N = 643	510 (79.3%)	133 (20.7%)	0.746
	Medium	N = 644	513 (79.7%)	131 (20.3%)	
	High	N = 640	518 (80.9%)	122 (19.1%)	
Distance to closest study site (km)*	<1	N = 699	679 (85.0%)	120 (15.0%)	<0.001
	≥1 and <3	N = 759	574 (75.6%)	185 (24.4%)	
	≥3	N = 369	288 (78.0%)	81 (22.0%)	
Type of illness*	Respiratory	N = 1123	892 (79.4%)	231 (20.6%)	<0.001
	General malaise, fever, or headache	N = 185	110 (59.5%)	75 (40.5%)	
	Wound/skin	N = 150	139 (92.7%)	11 (7.3%)	
	Gastrointestinal	N = 391	333 (85.2%)	58 (14.8%)	
	Other	N = 78	67 (85.9%)	11 (14.1%)	

*Statistically significant association with sector

Characteristics of medication usage were compared between the formal and informal sector (Table 3). Most medications purchased in the informal sector were orally administered (93.4%), and non-prescribed (78.8%). Antibiotic usage was common and used in 50.8% of all illness episodes. Antibiotic usage was less common for the informal sector than for the formal sector (29.3% vs 56.2%, p < 0.001, chi- square, Table 3). A secondary analysis considering pharmacies within the formal sector (S5 Table) found similar associations.

**Table 3:** Illness and medication use episode characteristics stratified by use of medication from the formal and informal sector using column percentages.

Variable	Category	Formal sector n (column %)	Informal sector n (column %)	P-value for chi-square test
**Medication use episodes**		**4663 (100%)**	**457 (100%)**	
Route of administration	Oral	3965 (85.0%)	427 (93.4%)	<0.001
	Parenteral	45 (1.0%)	0 (0%)	
	Topical	131 (2.8%)	5 (1.1%)	
	Nasal/otic	394 (8.4%)	5 (1.1%)	
	Ophthalmic	37 (0.8%)	5 (1.1%)	
	Inhaled	4 (0.1%)	1 (0.2%)	
	Missing	87 (1.9%)	14 (3.1%)	
Prescription	Yes	4504 (96.6%)	97 (21.2%)	<0.001
	No	159 (3.4%)	360 (78.8%)	
**Illness episodes**		**1541 (100%)**	**386 (100%)**	
Number of medications	1	646 (41.9%)	309 (80.1%)	<0.001
	>1	893 (57.9%)	76 (19.7%)	
	missing	2 (0.1%)	1 (0.3%)	
Antibiotic usage	No	675 (43.8%)	273 (70.7%)	<0.001
	Yes	866 (56.2%)	113 (29.3%)	

To understand how trial participation might influence formal and informal sector use and types of medication that were prescribed within the formal and informal sector we did a sensitivity analysis. When compared between the community control population and the clinical trial population, the control group visited the informal sector more often than the trial group for gastrointestinal disease (31.2% vs 11.6%; p < 0.001, chi-square, S3 Table)–a key clinical outcome for the vaccine trial participants. Whereas there was no significant difference in antibiotic usage from the informal sector between trial participants and community controls, antibiotic usage in the formal sector was more common for the community controls (79.8% vs 50.2%; p < 0.001, chi-square, S3 Table).

#### Type of illness, study site and distance are associated with informal medication use

Numerous variables were associated with informal sector medication use when evaluated using a mixed effects model (Table 4). Table 4 shows the adjusted odds ratios from the full logistic regression model with a random intercept for participant accounting for variability in the baseline probability of receiving medication from the informal sector per participant. Distance in kilometers to the closest study site was positively associated with use of medication from the informal sector (OR: 1.09; 95% CI: 1.01, 1.17). Participants enrolled in the community cohort (Chainda study site) had higher odds of use of medication from the informal sector than participants included at the Matero study site (OR: 3.18; 95% CI: 1.86, 5.46). Type of illness also associated with informal sector medication use with infants experiencing general malaise, fever, or headache having a higher odds of receiving medication from the informal health sector than infants suffering from respiratory disease (OR: 2.62; 95% CI: 1.75, 3.93), while infants experiencing wound/skin disease had lower odds of receiving medication from the informal health sector than infants suffering from respiratory disease (OR: 0.36; 95% CI: 0.18, 0.73). Gastrointestinal disease was not associated with use of medication from the informal sector (Table 4). A secondary analysis that considered pharmacies within the formal sector (S6 Table) found similar associations.

**Table 4:** Results of the logistic regression analysis with participant as a random intercept.

Child and illness episode characteristics	Adjusted odds ratio	95% CI
Sex		
Male	1	-
Female	0.99	0.69–1.44
Study site		
Matero (trial)	1	-
George (trial)	0.73	0.43–1.22
Chainda (control)	**3.18** ***	**1.86–5.46**
Socioeconomic status		
Low	1	-
Medium	0.63	0.40–1.02
High	0.80	0.51–1.25
Distance to closest study site (km)	**1.09** *	**1.01–1.17**
Type of illness		
Respiratory	1	-
General malaise, fever, headache	**2.62** ***	**1.75–3.93**
Wound/skin	**0.36** ***	**0.18–0.73**
Gastrointestinal	0.78	0.54–1.12
Other		
Mixed effects model parameters		
Marginal R^2^ / Conditional R^2^	0.124 / 0.357	

*p ≤ 0.05

***p ≤ 0.001

## Discussion

There is an urgent need to identify drivers of informal and non-prescription medication use, particularly for vulnerable patient populations such as pediatric populations in low- and middle-income settings that are at high risk for adverse outcomes from non-prescribed medications. This study, conducted in an infant cohort in Zambia, was able to identify several variables associating with informal sector medication use. Key among them were the type of illness a child had, the distance of the child’s home to the closest study clinic, and enrollment in a clinical trial. Illness episodes with general malaise, fever or headache were associated with the highest use of medication acquired in the informal sector. Parents living further away from the study clinic were more likely to obtain medication in the informal sector, and community controls were more likely to acquire medication in the informal sector compared to trial participants.

In our study, 20.0% of illness episode medications were acquired in the informal health sector. Previous studies have reported higher rates of (caregiver) self-medication, with proportions ranging from 39–79% depending on geographic and socioeconomic settings [15–19]. These medications are often acquired in pharmacies and local retail stories [20, 21], locations that we also defined as the informal health sector, since most medications acquired in these locations are non-prescribed. A secondary analysis including pharmacies as part of the formal sector found similar associations to the primary analysis. The cause of the lower reported informal health sector medication use in our study as compared to earlier published studies was likely due to the study structure where children were concomitantly enrolled in a clinical trial. Trial participants were actively stimulated to seek care at a formal health facility. Community cohort participants also had higher rates of informal medication usage than trial participants. Trial enrollment is therefore a likely confounder explaining the lower rate of informal medication use and self-medication in our study compared to studies in other low- and middle-income settings in sub-Saharan Africa.

The type of illness an infant had strongly predicted whether their caregiver would purchase medications in the informal sector. Illness episodes with general malaise, fever or headache were associated with the highest use of medication from the informal sector. It is possible that lower levels of illness severity for infants with general malaise, fever and headache contribute to this association. Previous research has shown that high-severity illness is associated with formal healthcare seeking behavior [22, 23], our study shows that low-severity illness may drive medication usage from the informal sector.

Trial participation and study characteristics may impact use of medication from the informal sector alongside illness characteristics and severity. We expected to find an association between gastrointestinal disease and use of medication from the informal sector, due to existing literature suggesting easy accessibility of medications for gastrointestinal events (e.g., oral rehydration solution) in the informal sector [24]. Gastrointestinal disease was not significantly associated with use of medication from the informal sector in our study. We found that 14.8% of gastrointestinal disease episodes were treated with medication from the informal sector, whereas previous research shows a proportion of approximately 30% [25, 26]. The lower-than expected use of medications from the informal sector for gastrointestinal episodes in our study might be biased because a large proportion of the study population participated in a vaccine trial aimed at preventing diarrhea, requiring them to visit the study clinic in case of illness, in particular for diarrheal symptoms. Evaluation of the community cohort alone (that did not participate in the trial) showed significantly higher informal sector medication use for gastrointestinal disease than the trial participants. This supports the assumption that trial participation biases the lack of association between gastrointestinal disease and informal sector medication usage.

Distance from the formal sector is also an important determinant of informal sector medication usage. The further an infant lived from a study clinic, the more likely they were to receive medication from the informal sector. This finding is logical, as travelling to a health facility costs time and money and can be inconvenient for caregivers. Previous research supports this finding [18, 27] Participation in the vaccine trial might have reduced the financial barriers to travelling to the formal sector, as transport to the clinic was arranged for trial participants. Therefore, the association between distance and use of medication from the informal sector is likely to have been underestimated. Our study does, however, suggest that overcoming financial barriers to accessing the formal sector can be an effective intervention for diminishing informal health care usage. Previous studies [28–31] have attempted to bridge geographic barriers by bringing the formal health care sector to patients and thereby decreasing travel costs. Mobile clinics, community health service delivery, and using mobile transport vouchers, have all proven to be effective interventions in improving formal care utilization [28–31].

Contrary to expectation, socioeconomic status (SES) was not associated with informal sector medication usage. Other studies have shown lower SES to be positively associated with use of medication from the informal sector [10, 27, 32]. Travelling to a formal health facility costs time and money, and it has been shown that individuals with the lowest SES live furthest away from health facilities [33]. Our finding might be biased by the fact that our study population had better access to healthcare than the general population in Zambia, because study participation reduced financial barriers to using formal health care facilities. It is reassuring that caregivers did not exhibit different treatment seeking behavior depending on the sex of their infant, previous literature in Nigeria and Yemen showed that male infants were more likely to receive treatment from the formal sector [34, 35].

Inappropriate antibiotic usage is one of the greatest risks of informal sector medication usage, as it is a key driver of antimicrobial resistance [36]. Despite high overall antibiotic usage rates among study participants, we found that antibiotic usage was less common in the informal sector than in the formal sector. In The Democratic Republic of Congo antibiotic use is less common in medicine stores than clinics and health centers [37], while in Ghana antibiotic use is more common in drug stores than hospitals [38]. To understand how trial participation might influence formal and informal sector use and types of medication that were prescribed within the formal and informal sector we did a sensitivity analyses. Utilization of the formal sector was higher among trial participants than in the control group. If infants sought care in the formal sector, antibiotic usage was lower in the trial participants. This might be caused by two factors: either by more low-severity illnesses in the formal setting among the trial group since these caregivers are stimulated to seek formal care, or by antibiotic prescribing being higher in clinics that are not participating in trials. It remains unclear whether low antibiotic usage from the informal sector in this study can be explained by the overarching study design providing good access to the formal sector. This could be clarified by qualitative research, and research evaluating the influence of illness severity and access to care on use of antibiotics from the formal sector.

There were many limitations to this study that are relevant to the interpretation of key study findings. The main limitation was that participation in the vaccine trial has influenced health-seeking behavior and medication use from the informal sector, because trial participants were stimulated and facilitated to seek care at the hospital or study clinic for their infant and sought more care in the formal sector than community controls, particularly for diarrheal symptoms. Another limitation, illustrated by the R-squared of the mixed effects model, is that we only explained a small proportion of the variance in use of medication from the informal sector. There might be other important characteristics associated with use of medication from the informal sector that we were unable to investigate, such as severity of the illness, educational status, age and marital profile of the caregiver, rural or urban household location, age of the child, number of children, season, HIV status, and nutritional status. A further limitation is that data were gathered through interviewer administered questionnaires and although research assistants were trained, this might have led to social desirability bias. Even though we tried to minimize the risk of recall bias by implementing a short recall period of one week and a medicine cabinet survey, recall bias cannot be ruled out [39]. A strength of our study is that household data came from a different source than most research performed on this topic. Data on consumption of medication, such as antibiotics, are scarce for LMICs [40]. Studies on illness and medication use are often based on data from large household surveys such as the Demographic and Health Surveys (DHS), which roughly estimates medication use and illness episodes on two-week recall by caregiver [23, 41, 42]. Our study is based on in-person weekly follow-up visits, providing more accurate information and a lower risk of recall bias.

In conclusion, our study provides essential information on key factors associated with use of medication from the informal sector by infants in an LMIC setting. Informal sector medication use is common and risk factors for obtaining medications in the informal sector included a long distance to a formal clinic, type and severity of illness, and not being enrolled in a clinical trial. Additional research is crucial to better identify the characteristics and motives driving caregivers to seek medications from the informal sector, and should include generalizable study populations, information on severity of disease, and an emphasis on qualitative research. Our findings suggest that improved access to formal health care services may decrease reliance on medication from the informal sector for infants. This study provides a strong foundation for designing and testing interventions that could significantly reduce geographic and knowledge barriers to accessing the formal health sector. Such evidence-based interventions are needed to protect young infants in LMICs from the threats of inappropriate medication use, including inappropriate antibiotic usage.

## Supporting information

S1 Table Prescription of medication in locations in the formal and informal health sector. (PDF)

S2 Table Location where medication was acquired in the informal health sector. (PDF)

S3 Table Medication use for gastrointestinal disease and antibiotic use within the formal and informal sector compared between the trial and control group. (PDF)

S4 Table Illness episode-level participant and illness characteristics stratified by use of medication from the formal and informal sector with pharmacy being considered formal sector. (PDF)

S5 Table Illness and medication use episode characteristics stratified by use of medication from the formal and informal sector with pharmacy being considered formal sector. (PDF)

S6 Table Results of the logistic regression analysis with participant as a random intercept with pharmacy being considered formal sector. (PDF)

S1 Text Questionnaire on inclusivity in global research. (PDF)

S2 Text STROBE statement. (DOCX)
