# Supplementary material for: Drivers of informal sector and non-prescription medication use in pediatric populations in a low- and middle-income setting: A prospective cohort study in Zambia
Source: PLOS Glob Public Health. 2023 Jul 6;3(7):e0002072. doi: 10.1371/journal.pgph.0002072 (PMC10325117; doi:10.1371/journal.pgph.0002072)
Supplement: S1 Table — (PDF) [file pgph.0002072.s001.pdf]

S1 Table. Prescription of medication in locations in the formal and informal health sector.

	Hospital (N=2003)	Community clinic (N=2657)	Pharmacy (N=284)	Street vendor (N=23)	Friend/relative/ neighbor (N=16)	Chemical shop (N=133)	Overall (N=5116)
Prescribed	1925 (96.1%)	2576 (97.0%)	47 (16.5%)	0 (0%)	3 (18.8%)	47 (35.3%)	4598 (89.9%)
Non-prescribed	78 (3.9%)	81 (3.0%)	237 (83.5%)	23 (100%)	13 (81.3%)	86 (64.7%)	518 (10.1%)
