# Supplementary material for: Drivers of informal sector and non-prescription medication use in pediatric populations in a low- and middle-income setting: A prospective cohort study in Zambia
Source: PLOS Glob Public Health. 2023 Jul 6;3(7):e0002072. doi: 10.1371/journal.pgph.0002072 (PMC10325117; doi:10.1371/journal.pgph.0002072)
Supplement: S2 Table — (PDF) [file pgph.0002072.s002.pdf]

S2 Table. Location where medication was acquired in the informal health sector.

Location	Total N=457 (100%)
Pharmacy	285 (62.4%)
Street vendor	23 (5.0%)
Friend/relative/neighbor	16 (3.5%)
Over the counter/Chemical shop	133 (29.1%)
