# Supplementary material for: Drivers of informal sector and non-prescription medication use in pediatric populations in a low- and middle-income setting: A prospective cohort study in Zambia
Source: PLOS Glob Public Health. 2023 Jul 6;3(7):e0002072. doi: 10.1371/journal.pgph.0002072 (PMC10325117; doi:10.1371/journal.pgph.0002072)
Supplement: S3 Table — (PDF) [file pgph.0002072.s003.pdf]

S3 Table. Medication use for gastrointestinal disease and antibiotic use within the formal and informal sector compared between the trial and control group.

Variable		Trial group n (column%)	Control group n (column%)	P-value for chi-square test
**Illness episodes with gastrointestinal disease**		**327 (100%)**	**64 (100%)**	
Formal sector		289 (88.4%)	44 (68.8%)	<0.001
Informal sector		38 (11.6%)	20 (31.3%)	
**Illness episodes with formal sector drug use**		1229 (100%)	312 (100%)	
Antibiotic usage	Yes	617 (50.2%)	249 (79.8%)	<0.001
	No	612 (49.8%)	63 (20.2%)	
**Illness episodes with informal sector drug use**		215 (100%)	171 (100%)	
Antibiotic usage	Yes	58 (27.0%)	116 (67.8%)	0.317
	No	157 (73.0%)	55 (32.2%)	
