# Supplementary material for: Drivers of informal sector and non-prescription medication use in pediatric populations in a low- and middle-income setting: A prospective cohort study in Zambia
Source: PLOS Glob Public Health. 2023 Jul 6;3(7):e0002072. doi: 10.1371/journal.pgph.0002072 (PMC10325117; doi:10.1371/journal.pgph.0002072)
Supplement: S4 Table — (PDF) [file pgph.0002072.s004.pdf]

S4 Table. Illness episode-level participant and illness characteristics stratified by use of medication from the formal and informal sector with pharmacy being considered formal sector.

Variable	Category	Total	Formal sector n (row %)	Informal sector n (row %)	P-value for chi-square test
**Illness episodes**		**N=1927**	**1773 (92.2%)**	**149 (7.8%)**	
Sex	Male	N=1000	905 (90.5%)	95 (9.5%)	0.003
	Female	N=927	873 (94.2%)	54 (5.8%)	
Study site*	Matero	N=429	407 (94.9%)	22 (5.1%)	<0.001
	George	N=1015	970 (95.6%)	45 (4.4%)	
	Chainda	N=483	401 (83.0%)	82 (17.0%)	
SES	Low	N=643	602 (93.6%)	41 (6.4%)	0.265
	Medium	N=644	592 (91.9%)	52 (8.1%)	
	High	N=640	584 (91.2%)	56 (8.8%)	
Distance to closest study site (km)	<1	N=799	747 (93.5%)	52 (6.5%)	0.002
	≥1 and <3	N=759	681 (89.7%)	78 (10.3%)	
	≥3	N=369	350 (94.9%)	19 (5.1%)	
Type of illness*	Respiratory	N=1123	1031 (91.8%)	92 (8.2%)	<0.001
	General malaise, fever, or headache	N=185	161 (87.0%)	24 (13.0%)	
	Wound/skin	N=150	147 (98.0%)	3 (2.0%)	
	Gastrointestinal	N=391	365 (93.4%)	26 (6.6%)	
	Other	N=78	72 (92.3%)	6 (7.7%)	

*Statistically significant association with sector
