# Supplementary material for: Drivers of informal sector and non-prescription medication use in pediatric populations in a low- and middle-income setting: A prospective cohort study in Zambia
Source: PLOS Glob Public Health. 2023 Jul 6;3(7):e0002072. doi: 10.1371/journal.pgph.0002072 (PMC10325117; doi:10.1371/journal.pgph.0002072)
Supplement: S5 Table — (PDF) [file pgph.0002072.s005.pdf]

S5 Table. Illness and medication use episode characteristics stratified by use of medication from the formal and informal sector with pharmacy being considered formal sector.

Variable	Category	Formal sector n (column %)	Informal sector n (column %)	P-value for chi-square test
**Medication use episodes**		**4948 (100%)**	**172 (100%)**	
Route of administration	Oral	4229 (85.5%)	168 (97.6%)	<0.001
	Parenteral	43 (0.9%)	0 (0%)	
	Topical	133 (2.7%)	0 (0%)	
	Nasal/otic	395 (8.0%)	1 (0.6%)	
	Ophthalmic	45 (0.9%)	0 (0%)	
	Inhaled	5 (0.1%)	0 (0%)	
	Missing	98 (2.0%)	3 (1.7%)	
Prescription	Yes	4552 (92.0%)	50 (29.1%)	<0.001
	No	396 (8.0%)	122 (70.9%)	
**Illness episodes**		**1773 (100%)**	**149 (100%)**	
Number of medications	1	831 (46.9%)	124 (83.2%)	<0.001
	>1	939 (53.0%)	25 (16.8%)	
	missing	3 (0.2%)	0 (0.0%)	
Antibiotic usage	No	856 (48.3%)	98 (65.8%)	<0.001
	Yes	917 (51.7%)	51 (34.2%)	
