# Supplementary material for: Drivers of informal sector and non-prescription medication use in pediatric populations in a low- and middle-income setting: A prospective cohort study in Zambia
Source: PLOS Glob Public Health. 2023 Jul 6;3(7):e0002072. doi: 10.1371/journal.pgph.0002072 (PMC10325117; doi:10.1371/journal.pgph.0002072)
Supplement: S6 Table — (PDF) [file pgph.0002072.s006.pdf]

S6 Table. Results of the logistic regression analysis with participant as a random intercept with pharmacy being considered formal sector.

Child and illness episode characteristics	Adjusted odds ratio	95% CI
Sex		
Male	1	-
Female	0.61	0.35-1.06
Study site		
Matero (trial)	1	-
George (trial)	1.03	0.45-2.34
Chainda (control)	**5.04*** **	**2.17-11.69**
Socioeconomic status		
Low	1	-
Medium	0.92	0.45-1.88
High	1.29	0.66-2.52
Distance to closest study site (km)	1.05	0.94-1.18
Type of illness		
Respiratory	1	-
General malaise, fever, headache	**2.44*** **	**1.32-4.50**
Wound/skin	**0.10*** **	**0.01-0.73**
Gastrointestinal	0.99	0.58-1.70
Other		
Mixed effects model parameters		
Marginal R^2 / Conditional R^2	0.167 / 0.488	

*p ≤ 0.05

***p ≤ 0.001
